# School-based prevalence and factors associated with depressive symptoms among secondary school adolescents in Mahagadhimai Municipality, Nepal

**DOI:** 10.1371/journal.pgph.0007225

**Published:** 2026-09-09

**Authors:** Harikishor Yadav, Dipendra Kumar Yadav, Mahesh Raj Bist, Nabin Bisht, Roshan Kumar Mahato

**Affiliations:** 1 School of Health and Allied Sciences, Faculty of Health Sciences, Pokhara University, Pokhara Metropolitan, Nepal; 2 Faculty of Public Health, Khon Kaen University, Khon Kaen, Thailand; 3 College of Medical Sciences, Kathmandu University, Bharatpur, Chitwan, Nepal; University of Balamand Faculty of Health Sciences, LEBANON

## Abstract

Depression is one of the most prevalent mental health disorders among adolescents globally and is a growing public health concern in Nepal. This study aimed to determine the school-based prevalence and factors associated with depressive symptoms among secondary school adolescents in Mahagadhimai Municipality, Nepal. An analytical cross-sectional study was conducted among 302 students in grades 11 and 12 from four public schools of Mahagadhimai Municipality, Madhesh Province, Nepal using multistage sampling. Depressive symptoms were assessed using the Centre for Epidemiological Studies Depression Scale (CES-D-20) via a self-administered questionnaire. Bivariate and multivariable logistic regression analysis were performed to identify the factors associated with depressive symptoms and results were presented as odds ratios (OR) with 95% CI. P < 0.05 was considered statistically significant. Nearly two-fifths of adolescents (39.70%) reported depressive symptoms, including 18.20% mild, 13.60% moderate and 7.90% severe depressive symptoms. Factors significantly associated with depressive symptoms included academic stress (AOR: 2.60, 95% CI: 1.48-4.56), problematic social media use (AOR: 2.93, 95% CI: 1.38-6.26), low support from friend (AOR: 2.14, 95% CI: 1.05-4.36) or other significant individuals (AOR: 2.09, 95% CI: 1.04-4.19). Poor help-seeking behavior such as keeping problems to oneself (AOR: 4.09, 95% CI: 2.19-7.64), not sharing concerns with parents (AOR: 2.21, 95% CI: 1.21-4.04) or friends (AOR: 2.51, 95% CI: 1.33-4.75) and low self-esteem (AOR: 2.16, 95% CI: 1.12-4.15). Sociodemographic characteristics showed no significant associations. Nearly two-fifths of secondary school adolescents reported depressive symptoms, which were independently associated with academic stress, problematic social media use, limited social support, poor help-seeking behavior and low self-esteem. While prospective studies are needed to establish causality, these findings highlight key psychosocial targets that should be prioritized in the development of future school-based mental health awareness and support strategies.

## Introduction

Depression is one of the most significant mental health disorders affecting adolescents in worldwide [1]. “It is a common mental disorder characterized by persistent low mood or loss of interest in usual activities for at least two weeks, accompanied by symptoms such as poor concentration, guilt, hopelessness, sleep or appetite changes, low energy, or suicidal thoughts, leading to impaired functioning” [2]. Currently, WHO estimates indicate that mental health conditions affect roughly 14.3% of the global adolescent population (ages 10–19), accounting for approximately one-seventh of disease burden in this demographic [3]. The Global Burden of Disease Study has consistently identified unipolar severe depression as a major contributor to disability-adjusted life years (DALYs) in 2020 [4].

Adolescence, typically defined as the period from 10 to 19 years of age, represents a developmental transition marked by significant physical, emotional and psychological changes from puberty through the onset of adulthood [5]. Exposure to violence, abuse, family conflict, academic pressure, or other stressful events can increase vulnerability to mental health problems, including depression [3,6]. Adolescent depression is a severe psychiatric disorder that impairs family, social and academic functioning [7]. If left unrecognized or untreated, depression may persist into adulthood, increasing the risk of major depressive episodes and other psychiatric disorders [8–10]. Addressing and preventing depression is essential for safeguarding adolescents from developing unhealthy behavior patterns.

By the year 2030, depression is expected to emerge as a significant global health issue, ranking third in disease burden for low-income nations and second for middle-income countries [11,12]. A comprehensive review and pooled analysis of 72 studies conducted between 2001 and 2020 found that approximately 34% of adolescents worldwide reported experiencing depressive symptoms at a given point in time [13]. In South Asia, school-based studies have reported high variability in adolescent depression, with prevalence rates of 52.2% in Iran [14], 49.2% in Bihar, India [15], 17.2% in Pakistan [16] and 26.5% in Dhaka Bangladesh [17]. Rather than reflecting simple geographical disparities, these variations stem from structural, socio-cultural and methodological differences. Methodologically, estimates fluctuate depending on the screening instruments employed (e.g., CES-D, PHQ-9, DASS-21) and the diagnostic cut-offs selected. Culturally, differences in mental health literacy, somatization and societal stigma strongly influence self-reporting behavior. Structurally, disparities in socio-economic stability, community resilience and healthcare access shape distinct risk profiles across populations. In Nepal, nearly one in four people are adolescents between 10 and 19 years old, representing roughly 24% of the population [18]. Despite this large proportion, few studies have addressed their psychosocial problems. In Nepal, the 2020 National Mental Health Survey reported that approximately 5.2% of adolescents aged 13–17 years experienced mental distress, with similar prevalence observed among both males and females [19]. However, localized investigations suggest considerably higher depression prevalence among school-enrolled adolescents. For instance, nearly half 44.2% of students in Pokhara Metropolitan reported depressive symptoms [20]; more than half 57.7% of adolescents in Harion Municipality, Sarlahi were affected [21] and 42.8% of adolescents in Birtamod Municipality, Jhapa, were identified as depressed using the CES-D scale [22]. Similarly, 55.6% of students in Tokha Municipality, Kathmandu, were found to have depressive symptoms using the DASS-21 [6], whereas a rural community-based study that utilized the PHQ-9 reported a lower prevalence of 27% [23].

**Fig 1:**
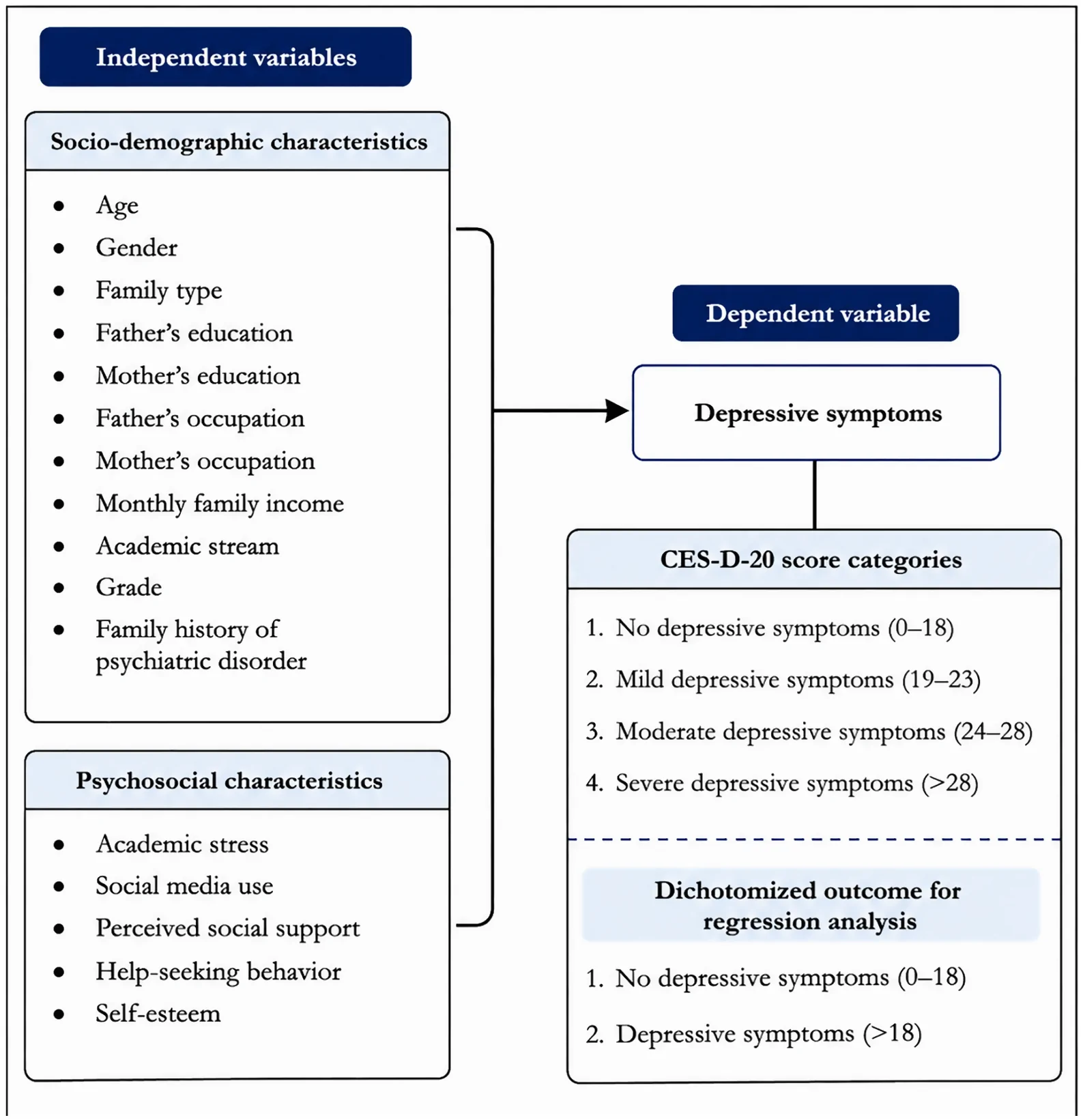
Conceptual Framework.

A critical gap remains in the literature regarding adolescent mental health in the rural and semi-urban southern plains of Nepal, specifically within Madhesh Province. Unlike metropolitan centers such as Kathmandu or Pokhara, Madhesh Province presents unique socio-cultural dynamics, socio-economic challenges and rapid digital adaptation, alongside limited mental health infrastructure. Mahagadhimai Municipality offers an important setting to study these dynamics, as local adolescents navigate high-stakes academic transitions (grades 11 and 12), evolving family roles and increasing digital media use. Without localized empirical evidence, public health authorities cannot design context-specific support strategies. To systematically examine adolescent mental health in this setting, this study was guided by a conceptual framework (Fig 1). The framework posits that an adolescent’s risk of developing depressive symptoms (assessed using the CES-D-20 scale) is influenced by sociodemographic characteristics (e.g., age, gender, family structure, parental education/occupation, income, Academic stream, grade and family psychiatric history) and psychosocial factors (academic stress, social media use, perceived social support, help-seeking behavior and self-esteem).Therefore, this study aimed to determine the school-based prevalence and factors associated with depressive symptoms among secondary school adolescents in Mahagadhimai Municipality, Nepal.

## Materials and methods

### Study design, population and setting

This cross-sectional analytical study was conducted among secondary school adolescents in grades 11 and 12, attending four public schools of Mahagadhimai (Bara District, Madesh Province, Nepal) from 7 August 2025–29 December 2025. According to 2021 national census data, the municipality encompasses 9,761 households with a total population of 59,421 individuals, including 13,040 adolescents aged 10–19 years [18].

### Sample size calculation and sampling technique

The sample size was calculated using Cochran’s formula for cross-sectional survey [24], n = z^2^ * p(1-p)/ e^2^. A prevalence of 25.3% for depression among adolescents, reported in a previous study conducted in Kathmandu, Nepal [25], was used as the reference value. Using a 95% confidence level and a 5% margin of error, the initial sample size was 291. After accounting for a 4% non-response rate, the final required sample size was 302.

A multistage proportional stratified random sampling method was employed to select participants. The study specifically focused on adolescents in grades 11 and 12, as this cohort represents a critical, high-stress academic transition involving national board examinations and future career trajectory decisions. Private schools in the municipality were excluded because they either had no active student enrollments in grades 11 and 12 at the time of data collection or were operational secondary programs were suspended due to insufficient student numbers. Consequently, an institutional census was applied across the municipality, including all four functional public secondary schools that offered grades 11 and 12.

Within these four public schools, participants were stratified by grade (Grade 11 and Grade 12) and academic stream (Management and Education). The overall target sample of 302 was allocated proportionally to each stratum (school × grade × stream) based on official enrollment records. Individual students within each stratum were subsequently chosen via simple random sampling using class attendance registers as the sampling frame. This sampling procedure ensured balanced and proportional representation of public-school students across grades and streams within Mahagadhimai Municipality.

### Data collection and Instruments section

Data were collected using a structured questionnaire developed based on the study objectives, comprising five self-administered sections. The first part gathered information on students’ sociodemographic characteristics and basic details about their schools. The second part included the Bergen Social Media Addiction Scale (BSMAS-6) [26], which measured the extent of students’ social media use. The third part used the Multidimensional Scale of Perceived Social Support (MSPSS-12) [27] to evaluate the level of support students perceived from family members, friends and significant others, along with questions related to their help-seeking behavior. The fourth part applied the Rosenberg Self-Esteem Scale (RES-10) [28] to assess students’ self-esteem level. The final section incorporated the Center for Epidemiologic Studies Depression Scale (CES-D-20) [29] to identify depressive symptoms among adolescents..

The CES-D instrument comprises 20 assessment items rated on a 4-point Likert scale 0 (‘rarely or never’) through 3 (‘most or constantly’), with four positively-worded items requiring reverse scoring during summation [29]. Total scores range from 0-60, with higher scores indicating higher levels of depressive symptoms. In the original version, a score of 16 or above indicates the presence of subthreshold depression [29]. However, a meta-analysis recommended a threshold cut-off 20 for better diagnostic, which showed improved sensitivity (0.86) and specificity (0.74) [30]. Validation studies of the Nepali version of the CES-D supported the use of a cut-off score of 18 and reported good internal consistency (Cronbach’s alpha = 0.82) [20]. Accordingly, the pre-tested translated Nepali version of the CES-D was used in this study, with a cut-off score of >18 applied to screen for depressive symptoms. Problematic social media use was measured via the 6-item Bergen Social Media Addiction Scale (BSMAS-6) on a 5-point Likert scale (1–5; total score 6–30), categorized into low/normal risk (6–17), moderate risk (18–24) and high risk (25–30) (Cronbach’s alpha = 0.88) [31]. Perceived social support was measured using the 12-item Multidimensional Scale of Perceived Social Support (MSPSS-12) on a 7-point Likert scale (1–7; total score 12–84), categorized into low (12–35), moderate (36–60) and high (61–84) support (Cronbach’s alpha = 0.88) [32]. Self-esteem was assessed using the 10-item Rosenberg Self-Esteem Scale (RES-10), scored on a 4-point scale (0–30 total score), categorized as low less than 15 or high more than 15 self-esteem (Cronbach’s alpha = 0.71) [33]. The (CES-D-20), (BSMAS-6), (MSPSS-12) and (RES-10) items showed acceptable internal consistency in this study, with a Cronbach’s alpha of 0.72, 0.73, 0.91 and 0.87.

### Data collection procedure and technique

Data collection was carried out after obtaining approval from the administrative section of the Mahagadhimai Municipality and the respective schools. The questionnaire, developed in Nepali, was pretested on approximately 10% of the intended (~32 students) from Maulapur Municipality to ensure clarity and relevance. Students were provided with self-administered questionnaires to complete anonymously in their classrooms and participation was entirely voluntary. An orientation session was provided to guide students on completing the questionnaire. Written informed consent was obtained from all participants and for those under 18 years, additional consent was obtained from their parents or guardians.

### Data analysis

The data were checked for completeness and consistency, cleaned, coded and entered into Epi-Data v3.1, and exported to SPSS v20 for analysis. Descriptive statistics were computed, with categorical variables expressed as frequencies and percentages and continuous variables presented as means and standard deviations.

Binary logistic regression was used to assess associations between depressive symptoms and independent variables. Unadjusted odds ratios (UOR) were obtained from individual regression models, and variables with p < 0.05 were entered into a multivariable binary logistic regression model to estimate adjusted odds ratios (AOR) with 95% confidence intervals.

### Ethical statement

This study was conducted in accordance with the ethical principles of the Declaration of Helsinki regarding research involving human participants. Ethical approval was obtained from the Institutional Review Committee (IRC) of Pokhara University (Reference No: IRC/34/2082/83). Formal permission was also obtained from Mahagadhimai Municipality and the management committees of the selected schools. Written informed consent was obtained from students aged 18 years and above, while for those under 18, parental or guardian written consent along with student written assent was collected. Participants were informed about the study objectives, voluntary participation and their right to withdraw at any time without consequences. Confidentiality was maintained by assigning unique identification codes and securely storing all data with restricted access. No incentives were provided; however, a brief mental health awareness session on stress management and coping strategies was offered after data collection.

## Results

Among 302 participants, three-fifths were female (59.90%) and more than two-thirds were aged ≥18 years (69.50%) reflects late school entry, grade repetition and interrupted schooling common in rural public schools within Madhesh Province. Most lived in joint families (59.30%). Two-fifths of fathers (41.70%) had basic education, while over half of mothers were illiterate (54.30%). Over half of fathers worked in agriculture (55.30%) and most mothers were housewives (79.50%). Nearly half of households earned NPR 20,000–50,000 (47.40%). Academically, over half studied Management (56.00%) and two-thirds were in Grade 12 (62.90%). A family history of mental illness was reported by one in seven (13.90%).

Regarding psychosocial factors, about half experienced academic stress 53.60%, half had no risk of social media addiction (47.00%), one-third were at risk (36.80%), and one-sixth problematic (16.20%). Three-fifths reported high family support (59.90%), two-fifths high friend/significant other support (38.40%-40.70%). Three-quarters preferred solving problems alone (73.8%) or sharing with parents/friends (72.20-75.80%). Three-quarters had high self-esteem (77.00%). These characteristics are shown in Table 1.

**Table 1:** Socio-demographic and psychosocial characteristics of participants (n = 302).

Socio-demographic and psychosocial characteristics		
Characteristics	Number (n)	Percentage (%)
**Gender**		
Male	121	40.10
Female	181	59.90
**Age (years)**		
<18	92	30.50
≥18	210	69.50
(Mean ± SD)	18.01 ± 1.04	
Range (min- max)	16-23	
**Family type**		
Nuclear	73	24.20
Joint	179	59.30
Extended	50	16.50
**Father’s education**		
Illiterate	35	11.60
Informal	47	15.60
Basic	126	41.70
Secondary	84	27.80
Bachelor and above	10	3.30
**Mother’s education**		
Illiterate	164	54.30
Informal	52	17.20
Basic	71	23.50
Secondary	15	5.00
**Father’s occupation**		
Business	14	4.60
Private Job	17	5.60
Daily wage/Labor	23	7.60
Foreign employment	32	10.60
Service	49	16.20
Agriculture	167	55.30
**Mother’s occupation**		
Daily wage/Labor	2	0.70
Private Job	2	0.70
Business	3	1.00
Service	4	1.30
Agriculture	51	16.90
House-wife	240	79.50
**Family monthly income (NPR)**		
<20,000	87	28.80
20,000-50,000	143	47.40
>50,000	72	23.80
**Academic stream**		
Education	133	44.00
Management	169	56.00
**Grade**		
Eleven	112	37.10
Twelve	190	62.90
**Family history of mental illness**		
Yes	42	13.90
No	260	86.10
**Psychosocial characteristics**		
**Perceived academic stress**		
No stressed (neutral, disagree and strongly disagree)	140	46.40
Stressed (agree and strongly agree)	162	53.60
**Social media addiction**		
No risk (Score 6–17)	142	47.00
At risk (score 18–23)	111	36.80
Problematic social media use (Score >23)	49	16.20
**Perceived social support**		
**Perceived family support**		
Low (mean score 1–3)	66	21.90
Moderate (mean score 3.1-5)	55	18.20
High (mean score > 5)	181	59.90
**Perceived friend support**		
Low (mean score 1–3)	49	16.20
Moderate (mean score 3.1-5)	137	45.40
High (mean score > 5)	116	38.40
**Perceived significant others Support**		
Low (mean score 1–3)	81	26.80
Moderate (mean score 3.1-5)	98	32.50
High (mean score > 5)	123	40.70
**Help-seeking behavior**		
**Problem-solving without sharing**		
Yes	223	73.80
No	79	26.20
**Share problems with parents**		
Yes	218	72.20
No	84	27.80
**Share problems with Friends**		
Yes	229	75.80
No	73	24.20
**Self-esteem**		
Low self-esteem (score < 15)	70	23.20
High self-esteem (score ≥ 15)	232	76.80

More than half of the participants (60.30%) had no depressive symptoms, while around one-fifth experienced mild (18.20%) or moderate (13.60%) depressive symptoms and less than one-tenth (7.90%) had severe depressive symptoms, indicating that nearly two-fifths of adolescents showed some level of depressive symptoms. These levels of depressive symptoms are presented in Table 2.

**Table 2:** Levels of depressive symptoms among school-going adolescents (n = 302).

Levels of depressive symptoms	Number (n)	Percentage (%)
No depressive symptoms (score 0–18)	182	60.30
Mild depressive symptoms (score 19–23)	55	18.20
Moderate depressive symptoms (score 24–28)	41	13.60
Severe depressive symptoms (score >28)	24	7.90

Among socio-demographic characteristics, family type was significantly associated with depressive symptoms, with adolescents from nuclear families had more than twice the odds of depressive symptoms compared with those from extended families (UOR: 2.36; 95% CI: 1.09-5.11; p = 0.028). No significant associations were observed for gender, age, parental education, parental occupation, monthly income, faculty, grade, or family history of mental illness.

All psychosocial factors were significantly associated with depressive symptoms. Adolescents with academic stress had nearly three times higher odds (UOR: 2.75; 95% CI: 1.69-4.46), and those with problematic social media use had more than four times higher odds (UOR: 4.40; 95% CI: 2.27-8.52). Adolescents with low or moderate family support had almost two times higher odds (UOR: 1.87; 95% CI: 1.16-2.99), low or moderate friend support had nearly three times higher odds (UOR: 2.68; 95% CI: 1.62-4.45), and low or moderate support from other significant persons had almost three times higher odds (UOR: 2.93; 95% CI: 1.77-4.84). Those who did not share problems with parents had nearly two times higher odds (UOR: 1.79; 95% CI: 1.07-2.98), did not share problems with friends had almost three times higher odds (UOR: 2.82; 95% CI: 1.64-4.86), and those with low self-esteem had about two times higher odds of depressive symptoms (UOR: 2.01; 95% CI: 1.17-3.45). The bivariate associations between socio-demographic and psychosocial characteristics and depressive symptoms are summarized in Table 3.

**Table 3:** Bivariate association between socio-demographic and psychosocial characteristics with depressive symptoms among school-going adolescents (n = 302).

Factors	Depressive symptoms		UOR	p-value
	Yes (%)	No (%)		
**Gender**				
Male	47 (38.80)	74 (61.20)	Ref	
Female	73 (40.30)	108 (59.70)	1.06 (0.66-1.70)	0.796
**Age**				
<18 years	44 (47.80)	48 (52.20)	1.61 (0.98-2.65)	0.058
≥18 years	76 (36.20)	134 (63.80)	Ref	
**Family type**				
Nuclear	35 (47.90)	38 (52.10)	2.36 (1.09-5.11)	0.028*
Joint	71 (39.70)	108 (60.30)	1.69 (0.85-3.35)	0.134
Extended	14 (28.00)	36 (72.00)	Ref	
**Father Education**				
Illiterate	16 (45.70)	19 (54.30)	1.36 (0.66-2.79)	0.398
Informal	20 (42.60)	27 (57.40)	1.20 (0.63-2.27)	0.577
Primary and above	84 (38.00)	136 (61.80)	Ref	
**Mother Education**				
Illiterate	63 (38.40)	101 (61.60)	Ref	
Informal	23 (44.20)	29 (55.80)	1.27 (0.67-2.39)	0.456
Primary and above	34 (39.50)	52 (60.50)	1.04 (0.61-1.78)	0.863
**Father Occupation**				
Agriculture	59 (35.30)	108 (64.70)	Ref	
Employed	61 (45.20)	74 (54.80)	1.50 (0.94-2.40)	0.082
**Mother Occupation**				
Agriculture	20 (39.20)	31 (60.80)	1.02 (0.55-1.90)	0.941
Employed	8 (61.50)	5 (38.50)	2.53 (0.80-7.99)	0.111
Housemaker	79 (46.50)	91 (53.50)	Ref	
**Monthly income**				
<20,000	39 (44.80)	48 (55.20)	1.43 (0.75-2.72)	0.267
20,000-50,000	55 (38.50)	88 (61.50)	1.10 (0.61-1.98)	0.737
>50,000	26 (36.10)	46 (63.90)	Ref	
**Academic stream**				
Education	59 (44.40)	74 (55.60)	1.41 (0.88-2.24)	0.146
Management	61 (36.10)	108 (63.80)	Ref	
**Grade**				
Eleven	40 (35.70)	72 (64.30)	Ref	
Twelve	80 (42.10)	110 (57.90)	1.30 (0.80-2.12)	0.273
**Family history of mental illness**				
Yes	18 (42.90)	24 (57.10)	1.16 (0.60-2.24)	0.656
No	102 (39.20)	158 (60.80)	Ref	
**Psychosocial characteristics**				
**Perceived academic Stress**				
Not stressed	38 (27.10)	102 (72.90)	Ref	
Stressed	82 (50.60)	80 (49.40)	2.75 (1.69-4.46)	<0.001*
**Social media addiction**				
No risk	86 (34.00)	167 (66.00)	Ref	
At risk/ problematic social media use	34 (69.40)	15 (30.60)	4.40 (2.27-8.52)	<0.001*
**Perceived social support**				
**Perceived Family Support**				
Low and moderate	59 (48.80)	62 (51.20)	1.87 (1.16-2.99)	0.009*
High	61 (33.70)	120 (66.30)	Ref	
**Perceived Friend Support**				
Low and moderate	90 (48.40)	96 (51.60)	2.68 (1.62-4.45)	<0.001*
High	30 (25.90)	86 (74.10)	Ref	
**Perceived other significant Support**				
Low and moderate	89 (49.70)	90 (50.30)	2.93 (1.77-4.84)	<0.001*
High	31 (25.20)	92 (74.80)	Ref	
**Help-seeking behavior**				
**Problem-solving without sharing**				
Yes	71 (31.80)	152 (68.20)	Ref	<0.001*
No	49 (62.0)	30 (38.0)	3.49 (2.04-5.96)	
**Share problems with parents**				
Yes	78 (35.80)	140 (64.20)	Ref	
No	42 (50.00)	42 (50.00)	1.79 (1.07-2.98)	0.024*
**Share problems with friends**				
Yes	77 (33.60)	152 (64.40)	Ref	
No	43 (58.90)	30 (41.10)	2.82 (1.64-4.86)	<0.001*
**Self-esteem**				
Low self-esteem	37 (52.90)	33 (47.10)	2.01 (1.17-3.45)	0.011*
High self-esteem	83 (35.80)	149 (64.20)	Ref	

Multicollinearity was checked using the Variance Inflation Factor (VIF), and all variables demonstrated VIF values below 5, indicating no multicollinearity concerns. Table 4 presents the predictors of depressive symptoms among adolescents using binary logistic regression. The model, adjusted for all variables, demonstrated good fit (Hosmer-Lemeshow χ² = 2.995, P = 0.935) and moderate explanatory power (Cox & Snell R^2^ = 0.264; Nagelkerke R^2^ = 0.357), with statistical significance set at p < 0.05.

**Table 4:** Multivariable logistic regression analysis of factors associated with depressive symptoms among school-going adolescents (n = 302).

Factors	Depressive symptoms		AOR (95% CI)	p-value
	Yes (%)	No (%)		
**Perceived academic Stress**				
Not stressed	38 (27.10)	102 (72.90)	Ref	
Stressed	82 (50.60)	80 (49.40)	2.60 (1.48-4.56)	<0.001*
**Social media addiction**				
No risk	86 (34.00)	167 (66.00)	Ref	
At risk/problematic social media use	34 (69.40)	15 (30.60)	2.93 (1.38-6.26)	0.005*
**Perceived social support**				
**Perceived Friend Support**				
Low and moderate	90 (48.40)	96 (51.60)	2.14 (1.05-4.36)	0.035*
High	30 (25.90)	86 (74.10)	Ref	
**Perceived other significant Support**				
Low and moderate	89 (49.70)	90 (50.30)	2.09 (1.04-4.19)	0.036*
High	31 (25.20)	92 (74.80)	Ref	
**Help-seeking behavior**				
**Problem-solving without sharing**				
Yes	71 (31.80)	152 (68.20)	Ref	
No	49 (62.00)	30 (38.00)	4.09 (2.19-7.64)	<0.001*
**Share problems with parents**				
Yes	78 (35.80)	140 (64.20)	Ref	
No	42 (50.00)	42 (50.00)	2.21 (1.21-4.04)	0.010*
**Share problems with Friends**				
Yes	77 (33.60)	152 (64.40)	Ref	
No	43 (58.90)	30 (41.10)	2.51 (1.33-4.75)	0.005*
**Self-esteem**				
Low self-esteem	37 (52.90)	33 (47.10)	2.16 (1.12-4.15)	0.020*
High self-esteem	83 (35.80)	149 (64.20)	Ref	

*Significance at p < 0.05; AOR: adjusted odds ratio; UOR: unadjusted odds ratio.

#Logistic regression model adjusted for all variables in the table, Cox & Snell R^2^ = 0.264, Nagelkerke R^2^ = 0.357 and Hosmer-Lemeshow χ2 = 2.995, P = 0.935.

In the adjusted analysis, adolescents experiencing academic stress had more than twice the odds of depressive symptoms compared with those without stress (AOR: 2.60; 95% CI: 1.48-4.56), and those with problematic social media use had nearly three times higher odds (AOR: 2.93; 95% CI: 1.38-6.26). Adolescents with low or moderate friend support (AOR: 2.14; 95% CI: 1.05-4.36) or low or moderate support from other significant persons (AOR: 2.09; 95% CI: 1.04-4.19) were about twice as likely to experience depressive symptoms as those with high support. Regarding help-seeking behaviors, adolescents who did not usually try to think about their problems and find solutions by themselves without sharing them with others had significantly four times higher odds of depressive symptoms than those who reported doing so (AOR = 4.09, 95% CI: 2.19–7.64), while those who did not share problems with parents (AOR: 2.21; 95% CI: 1.21-4.04) or friends (AOR: 2.51; 95% CI: 1.33-4.75) had about two- to two-and-a-half times higher odds. Low self-esteem was also a significant predictor, with affected adolescents having approximately twice the odds of depressive symptoms compared with those reporting high self-esteem (AOR: 2.16; 95% CI: 1.12-4.15). These multivariable analysis results are shown in Table 4.

## Discussions

This study identified a substantial prevalence of depressive symptoms (39.70%) among secondary school students in Mahagadhimai Municipality. The findings align with school-based research conducted in Nepal, such as the 42.80% prevalence reported by Giri et al. in Birtamod Municipality using the CES-D scale [22]. This results are slightly higher than the 27.5% reported in an urban area of Kathmandu [34] and slightly lower than the 57.7% observed among secondary school students in rural areas of Sarlahi [21].

Notably, this school-based estimate is considerably higher than the 5.2% prevalence of mental distress reported among Nepalese adolescents aged 13–17 years in the National Mental Health Survey (2020) [35]. However, numerous school-based studies in Nepal have reported substantially higher prevalence estimates, ranging from 13.1% to 57.7% [21,23,36,37]. This discrepancy likely reflects differences in study populations and settings rather than measurement alone. National household surveys include a broader adolescent population, whereas school-based studies capture adolescents experiencing academic evaluation, peer comparison, and examination-related pressures factors that were significantly associated with depressive symptoms in the present study.

International comparisons further confirm that elevated depressive symptoms among school-attending adolescents are a global phenomenon: 49.2% in Bihar, India [15], 57.7% in North Kerala, India [38], 52.6% in Iran [14], 26.5% in Bangladesh [17], 13.03% in China [39], 68.9% in Ghana [40] and 28% in Southwest, Ethiopia [41]. The substantial prevalence observed in this study, comparable to other South Asian contexts, reinforces that adolescent depressive symptoms represent a significant public health concern requiring urgent attention, particularly in settings like this one where school-based mental health screening and support infrastructure remains limited.

### Association between socio-demographic factors and depressive symptoms

Socio-demographic characteristics including age, gender, family structure, parental education and occupation, income level, faculty, academic grade and family psychiatric history were not significantly associated with depressive symptoms. These findings are consistent with studies from Nepal and neighbouring countries [15,17,20,22]. This pattern suggests that depressive symptoms in this population are not concentrated in a demographically identifiable subgroup, but instead cut across socio-demographic lines. Practically, this indicates that psychosocial and behavioural factors rather than background characteristics are what distinguish adolescents at higher risk, which has implications for how school-based screening should be targeted: broadly, rather than toward specific demographic profiles.

### Factors associated with depressive symptoms among school adolescents

Regarding psychosocial factors, academic stress was significantly associated with depressive symptoms: students experiencing academic stress had more than twice the likelihood of reporting depressive symptoms. This is consistent with findings from Birtamod Municipality, Jhapa [22] and research conducted by Karki et al. [6]. In the Nepali secondary-school system, Grades 11–12 mark the transition toward board examinations and career-track decisions, a period when academic performance is closely tied to family expectations and future socioeconomic mobility. This concentration of stakes within a single high-pressure window may explain why academic stress emerged as one of the strongest correlates in our sample, consistent with a stress-vulnerability pathway in which sustained evaluative pressure, without adequate coping resources, contributes to depressive symptoms.

Problematic social media use was similarly associated with depressive symptoms, with adolescents reporting problematic use nearly three times more likely to experience depressive symptoms. This is consistent with evidence from Kathmandu, Nepal [42] and Ethiopia [43]. Problematic use characterized by preoccupation and loss of control rather than time spent alone may displace sleep and in-person peer interaction while increasing exposure to social comparison, a combination plausibly compounding the academic pressures already present in this age group.

Perceived social support was strongly associated with depressive symptoms. Adolescents with low or moderate support from friends or other significant individuals had roughly twice the odds of depressive symptoms compared with those with high support, consistent with evidence from Pokhara Metropolitan City [20], Birtamod Municipality [22] and a Finnish longitudinal study [44]. This association is plausible given that supportive relationships with friends and significant others can act as a buffer against the academic and social stressors adolescents in this age group commonly face, whereas adolescents lacking such support have fewer external resources to draw on when coping with distress.

Help-seeking behaviour also showed a strong association with depressive symptoms: adolescents who did not discuss their problems with parents or friends had approximately two to two-and-a-half times higher odds of depressive symptoms, consistent with patterns reported in Pokhara Metropolitan City and Birtamod Municipality [20,22] and qualitative findings from India [45]. This finding is closely related to the social support results above, but captures a distinct behavioural dimension: even when support may be available, adolescents may not disclose their difficulties, whether due to stigma around discussing emotional problems, fear of judgment, or a learned preference for self-reliance. In the Nepali context, where expressing personal or emotional difficulties may be perceived as a sign of weakness, this reluctance to seek help may leave adolescents to manage distress alone even within otherwise supportive environments, compounding their vulnerability to depressive symptoms.

Self-esteem was also significantly associated with depressive symptoms: adolescents with low self-esteem had about twice the odds of depressive symptoms, consistent with studies from Birtamod Municipality, Jhapa [22], Pokhara, Nepal [20] and Vietnam, where adolescents with low self-esteem had almost fivefold higher odds of depressive symptoms [46]. Low self-esteem may reduce adolescents’ perceived capacity to cope with academic and social stressors and increase vulnerability to negative self-evaluation, compounding rather than acting independently of the other factors identified here.

In contrast, perceived family support did not remain significantly associated with depressive symptoms in the adjusted model. This may reflect shared variance with friend and other-significant-person support, given that during adolescence, peer relationships often become a more salient source of emotional support than family relationships, potentially attenuating the independent contribution of family support once these overlapping sources are accounted for. Overall, the findings indicate that academic stress, problematic social media use, limited help-seeking behaviour, low perceived support from friends or other significant individuals and low self-esteem are the factors most strongly associated with depressive symptoms among adolescents in this population.

### Strengths and limitations

This study has some limitations. Its cross-sectional design does limit the ability to establish cause-and-effect relationships between depressive symptoms and its associated factors. Data were collected using self-reported questionnaires, which may be associated by recall or social desirability bias, particularly for sensitive issues such as depressive symptoms and social media addiction. The study was conducted only among public secondary school students from a single municipality in Bara District, Madhesh Province, so the findings may not apply to adolescents in other areas, private schools, or those out of school. Additionally, depressive symptoms were measured using the CES-D screening tool rather than a clinical diagnostic method, which could affect accuracy. Important factors such as substance use, bullying, traumatic experiences and chronic illnesses were not assessed and psychosocial variables were measured at a single point in time. The focus on grades 11 and 12 also excludes younger adolescents who may face different risks. Despite these limitations, the study provides useful insights into adolescent mental health in Madhesh Province and highlights the need for larger, more diverse, and long-term studies.

## Conclusion

Nearly two-fifths of school adolescents reported depressive symptoms, primarily associated by academic stress, problematic social media use, limited social support, poor help-seeking behaviours, and low self-esteem. These findings highlight academic stress, social media use, social support, help-seeking behaviour and self-esteem as potentially modifiable factors that warrant consideration in school-based mental health promotion and early psychosocial support efforts, alongside referral pathways for at-risk students to clinical assessment. Given the cross-sectional design of this study, longitudinal and intervention research is needed to determine whether programs addressing these factors such as stress management, healthy social media use, self-esteem building, peer support and safe-to-talk spaces are effective in improving adolescent emotional well-being.

## Supporting information

S1 Data Minimal anonymized quantitative dataset containing respondent demographics, survey scores and regression variables underlying the study findings (also deposited in Zenodo at https://doi.org/10.5281/zenodo.21805403). (SAV)

S1 Text English language versions of the structured questionnaire used for assessing adolescent depressive symptoms, sociodemographic factors and psychosocial support mechanisms. (DOCX)
