# Supplementary material for: School-based prevalence and factors associated with depressive symptoms among secondary school adolescents in Mahagadhimai Municipality, Nepal
Source: PLOS Glob Public Health. 2026 Sep 9;6(9):e0007225. doi: 10.1371/journal.pgph.0007225 (PMC13557374; doi:10.1371/journal.pgph.0007225)
Supplement: Supplementary file 1 [file pgph.0007225.s002.docx]

**Part I: Sociodemographic And Academic Related Information**

| Q.N. | Questions | Response options |
| --- | --- | --- |
| A1 | Sex | 1. Male 2. Female 3. Other |
| A2 | Age | ____________ (write actual age in years) |
| A3 | Family type | 1. Nuclear (parents, son /daughter) 2. Joint (parents, grandparents, son/daughter) 3. Extended (parents, grandparents, son/daughter, uncles/aunts/cousins) |
| A4 | Father’s education | 1. Illiterate 2. No formal education (Can only read and write in Nepali) 3. Basic (5-8) 4. Secondary (9-12) 5. Bachelor and above |
| A5 | Mother’s education | 1. Illiterate 2. No formal education (Can only read and write in Nepali) 3. Basic (5-8) 4. Secondary (9-12) 5. Bachelor and above |
| A6 | Father’s occupation | 1. Agriculture 2. Foreign employment 3. Government job 4. Business or self-employed 5. Private or NGOs/INGOs sector 6. Daily wage/labour 7. Unemployed 8. Other (if other specify) ____________ |
| A7 | Mother’s occupation | 1. Agriculture 2. Foreign employment 3. Government job 4. Business or self-employed 5. Private or NGOs/INGOs sector 6. Daily wage/labour 7. Unemployed 8. Housewife 9. Other (if other specify) ____________ |
| A8 | Monthly family income | ______________ (write in Nepali rupees) |
| A9 | Family history of psychiatric disorder | 1. Yes 2. No |
| A10 | Which stream do you study? | 1. Science 2. Management 3. Education 4. Technical 5. Others (if others then specify) ___________ |
| A11 | In which class do you study? | 1. Eleven 2. Twelve |
| **Academic stress** | | |
| A12 | On a scale of 1-5 how much do you agree with the following statement.  **“I feel very stressed due to my studies.”** | 1. Strongly agree 2. Agree 3. Neutral 4. Disagree 5. Strongly disagree |

**Part II: Bergen Social Media Addiction Related Questions**

| The following statements refer to your experiences with social media use. Please tick (✓) the number that best represents how often each statement applies to you over the past year. | | | | | | |
| --- | --- | --- | --- | --- | --- | --- |
| Q.N. | Questions | 1(Very rarely) | 2(Rarely) | 3(Sometimes) | 4(Often) | 5(Very often) |
| B1 | You spend a lot of time thinking about social media or planning how to use it. |  |  |  |  |  |
| B2 | You feel an urge to use social media more and more. |  |  |  |  |  |
| B3 | You use social media in order to forget about personal problems. |  |  |  |  |  |
| B4 | You have tried to cut down on the use of social media without success. |  |  |  |  |  |
| B5 | You become restless or troubled if you are prohibited from using social media. |  |  |  |  |  |
| B6 | You use social media so much that it has had a negative impact on your job/studies. |  |  |  |  |  |

**Part III: Multidimensional Scale of Perceived Social Support Related Questions**

| The following statements are related to the support you receive from different people in your life. For each statement, please indicate how much you agree or disagree by ticking (√) one box on the scale of 1 to 7. | | | | | | | | |
| --- | --- | --- | --- | --- | --- | --- | --- | --- |
| Q.N. | statements | 1 (Very Strongly Disagree) | 2 (Strongly Disagree) | 3 (Mildly Disagree) | 4 (Neutral) | 5 (Mildly Agree) | 6 (Strongly Agree) | 7 (Very Strongly Agree) |
| C1 | My family really tries to help me. |  |  |  |  |  |  |  |
| C2 | I get the emotional help and support I need from my family |  |  |  |  |  |  |  |
| C3 | I can talk about my problems with my family. |  |  |  |  |  |  |  |
| C4 | My family is willing to help me make decisions. |  |  |  |  |  |  |  |
| C5 | My friends really try to help me. |  |  |  |  |  |  |  |
| C6 | I can count on my friends when things go wrong. |  |  |  |  |  |  |  |
| C7 | I have friends with whom I can share my joys and sorrows. |  |  |  |  |  |  |  |
| C8 | I can talk about my problems with my friends. |  |  |  |  |  |  |  |
| C9 | There is a special person who is around when I am in need. |  |  |  |  |  |  |  |
| C10 | There is a special person with whom I can share my joys and sorrows. |  |  |  |  |  |  |  |
| C11 | I have a special person who is a real source of comfort to me. |  |  |  |  |  |  |  |
| C12 | There is a special person in my life who cares about my feelings. |  |  |  |  |  |  |  |

**Part IV: Help-Seeking Behaviour Related Questions**

| Please read each statement carefully and tick (✓) the most appropriate answer. | | |
| --- | --- | --- |
| Q.N. | Statements | Response |
| D1 | Do you usually try to think about your problems and find solutions by yourself without sharing them with others? | 1. Yes 2. No |
| D2 | Do you share your problems with your parents? | 1. Yes 2. No |
| D3 | Do you share your problems with your friends? | 3. Yes 4. No |

**Part V: Rosenberg Self Esteem Related Questions**

| Below is a list of statements dealing with your general feelings about yourself. Please indicate how strongly you agree or disagree with each statement by selecting one for four options | | | | | |
| --- | --- | --- | --- | --- | --- |
| Q.N. | Statements | 3 (Strongly Agree) | 2 (Agree) | 1 (Disagree) | 0 (Strongly Disagree) |
| E1 | On the whole, I am satisfied with myself |  |  |  |  |
| E2 | At times, I think I am no good at all |  |  |  |  |
| E3 | I feel that I have a number of good qualities |  |  |  |  |
| E4 | I am able to do things as well as most other people |  |  |  |  |
| E5 | I feel I do not have much to be proud of |  |  |  |  |
| E6 | I certainly feel useless at times |  |  |  |  |
| E7 | I feel that I'm a person of worth, at least on an equal plane with others |  |  |  |  |
| E8 | I wish I could have more respect for myself. |  |  |  |  |
| E9 | All in all, I am inclined to feel that I am a failure |  |  |  |  |
| E10 | I take a positive attitude toward myself |  |  |  |  |

**Part VI: Center For Epidemiologic Studies Depression Scale Related Questions**

| Below is a list of the ways you might have felt or behaved. Please indicate how often you have felt this way during the past week | | | | | |
| --- | --- | --- | --- | --- | --- |
| Q.N. | Statements | 0 (Rarely or none of the time (less than 1 day) | 1 (Some or a little of the time (1–2 days) | 2 (Occasionally or a moderate amount of time (3–4 days) | 3 (Most or all of the time (5-7 days) |
| F1 | I was bothered by things that usually don’t bother me. |  |  |  |  |
| F2 | I did not feel like eating; my appetite was poor. |  |  |  |  |
| F3 | I felt that I could not shake off the blues even with help from my family or friends. |  |  |  |  |
| F4 | I felt I was just as good as other people. |  |  |  |  |
| F5 | I had trouble keeping my mind on what I was doing. |  |  |  |  |
| F6 | I felt depressed. |  |  |  |  |
| F7 | I felt that everything I did was an effort. |  |  |  |  |
| F8 | I felt hopeful about the future. |  |  |  |  |
| F9 | I thought my life had been a failure. |  |  |  |  |
| F0 | I felt fearful. |  |  |  |  |
| F11 | My sleep was restless. |  |  |  |  |
| F12 | I was happy. |  |  |  |  |
| F13 | I talked less than usual. |  |  |  |  |
| F14 | I felt lonely. |  |  |  |  |
| F15 | People were unfriendly. |  |  |  |  |
| F16 | I enjoyed life. |  |  |  |  |
| F17 | I had crying spells. |  |  |  |  |
| F18 | I felt sad. |  |  |  |  |
| F19 | I felt that people dislike me. |  |  |  |  |
| F20 | I could not get “going.” |  |  |  |  |

Thank you for your participants!
