# Supplementary material for: School-based prevalence and factors associated with depressive symptoms among secondary school adolescents in Mahagadhimai Municipality, Nepal
Source: PLOS Glob Public Health. 2026 Sep 9;6(9):e0007225. doi: 10.1371/journal.pgph.0007225 (PMC13557374; doi:10.1371/journal.pgph.0007225)
Supplement: Supplementary file 2 [file pgph.0007225.s003.docx]

**RESPONSE TO REVIEWERS**

**Manuscript ID:** PGPH-D-26-01271
**Title:** School-based Prevalence and Factors Associated with Depression among Secondary School Adolescents in Nepal

**Dear Editors and Reviewers,**

We sincerely thank the reviewers and the Academic Editor for their valuable comments and suggestions. We have carefully revised the manuscript accordingly. All changes have been incorporated in the revised manuscript, and a track changes version has also been submitted for transparency.

**Comment 1: Ethical approval and documentation**

Please clarify ethical approval and provide necessary supporting documents (e.g., IRC approval, consent procedures, and study instruments).

**Response:**

Thank you for this important comment. We have revised the manuscript to clearly report the ethical approval details. The Institutional Review Committee (IRC) approval number has now been added in the Ethical Considerations section of the manuscript.

In addition, we have uploaded the following supporting documents:

1. Ethical approval letter from the Institutional Review Committee (IRC) and Municipality
2. Completion letters from the respective schools
3. Complete survey instrument used for data collection, including informed consent and assent forms

We have also carefully revised the entire manuscript to address all reviewer and editor comments, improving clarity, language, and structure. All required documentation has been completed and submitted as part of this resubmission package.

**Closing Statement**

We hope that the revised manuscript meets the journal’s requirements and is now suitable for publication. We sincerely appreciate the time and effort of the editors and reviewers in improving our work.

Sincerely,
Harikishor Yadav
(On behalf of all authors)
School of Health and Allied Sciences
Pokhara University, Nepal
Email: hariyadavph@gmail.com
