# Supplementary material for: School-based prevalence and factors associated with depressive symptoms among secondary school adolescents in Mahagadhimai Municipality, Nepal
Source: PLOS Glob Public Health. 2026 Sep 9;6(9):e0007225. doi: 10.1371/journal.pgph.0007225 (PMC13557374; doi:10.1371/journal.pgph.0007225)
Supplement: Supplementary file 3 [file pgph.0007225.s004.docx]

PGPH-D-26-01342 comments

The topic is interesting and relevant and findings have potential implications on the mental wellbeing of adolescents. The below comments should be taken into consideration to improve the quality of the manuscript to become ready for publication.

The title should be focused on the area where the study took place to avoid providing an inflated title and misleading the reader.

The CES-D is a screening tool of the depressive symptoms and not depression. Please correct this throughout the entire document.

Introduction

It would be better if you integrate a conceptual framework that guides the study.

Explain why the prevalence of depression differs between countries. This would provide an analytical rather than descriptive aspect of the literature review.

The rationale of the study is very briefly highlighted. You have to provide more evidence about the gaps in the literature and significance of the study within its context, Mahagadhimai Municipality, Nepal.

Methods

Justify why the sample was limited only to adolescents in grades 11 and 12, attending four public schools.

You said that all four public secondary schools in Mahagadhimai Municipality that provided Grades 11 and 12 were included through a census approach. What do you mean by census, as these are the only four schools?

The sample is limited to a particular category of the population in a particular area, which limits the generalisation of findings. This limitation should be added in the discussion.

Why the private schools were excluded from the study?

“academic faculty” is confusing for the non-Nepalese readers.

The (CES-D-20) is the only described tool and all the others are neglected (BSMAS-6), (MSPSS-12) and (RES-10).

No evidence of validity or reliability is provided of the academic stress measurement.

Help-seeking variables are simplistic. Variables such as share problems with parents and share problems with friends appear to be single-item measures. There is no evidence of validity or reliability.

Statistical analysis

Include in multivariable analysis all variables with p < 0.25 in bivariate analysis to account with the biggest number of potential confounders.

Family support was significant in bivariate analysis but not included in the multivariate analysis model.

Justify why self-esteem, social support, and social media use were collapsed into categories, which limit their power.

Results

Depression prevalence appears lower among those who keep problems to themselves than among those who do not. Yet the discussion interprets the opposite.

69.5% were ≥18 years old.

For grades 11–12 this seems somewhat high and requires explanation

Table 1 states that the Median (min-max) age is 16–23. But this is not a median.

Justify why in the regression model, you collapsed the support into low + moderate categories

Discussion

The discussion is descriptive rather than analytical with many redundant ideas.

Avoid the overuse of predictors a this is a cross-sectional study

It is very common to

Family support lost significance in the adjusted model. This is theoretically important and deserves discussion.

The conclusion implies intervention effectiveness which is not an accurate terminology for a cross-sectional study.
