# Supplementary material for: School-based prevalence and factors associated with depressive symptoms among secondary school adolescents in Mahagadhimai Municipality, Nepal
Source: PLOS Glob Public Health. 2026 Sep 9;6(9):e0007225. doi: 10.1371/journal.pgph.0007225 (PMC13557374; doi:10.1371/journal.pgph.0007225)
Supplement: Supplementary file 4 [file pgph.0007225.s005.docx]

**Response to Reviewers**

**Manuscript ID:** PGPH-D-26-01342

**Title:** “School-based Prevalence and Factors Associated with Depressive symptoms among Secondary School Adolescents in Mahagadhimai Municipality, Nepal”

**Journal:** PLOS Global Public Health

**Dear Dr. Mathilde Azar and Reviewers,**

We would like to thank the Academic Editor and the reviewers for their time, constructive feedback and valuable insights regarding our manuscript. We have carefully addressed each point raised and we believe these revisions have significantly strengthened the quality, clarity and rigor of our paper.

Below, we provide a point-by-point response detailing how each comment has been addressed. The corresponding changes have been highlighted in the “**Revised Manuscript with Track Changes”** file.

**Part 1: Journal Requirements**

**Requirement 1: Financial Disclosure Statement**

**Comment:** Please provide a/amend your detailed Financial Disclosure statement... Please clarify all sources of funding...

- **Response:** We have updated our Financial Disclosure Statement to meet the journal's exact requirements.
- **Action taken:**

“The authors received no specific funding for this work”.

**Requirement 2: Data Availability Statement**

**Comment:** We note that you have indicated that there are restrictions to data sharing for this study... Please address ethical/legal restrictions or provide repository details.

- **Response:** We have updated our Data Availability Statement to comply fully with the PLOS Data Availability Policy. While our initial submission noted potential restrictions regarding minor participants, we have prepared a fully anonymized minimal dataset that strips all direct and indirect identifiers, adhering to the ethical mandates of our Institutional Review Committee (IRC). This minimal dataset has now been deposited in a public repository without restrictions.
- **Action taken:** The fully anonymized minimal dataset underlying all tables, figures, and statistical analyses in this manuscript has been deposited in **Zenodo** and is publicly accessible.
- **Repository Name:** Zenodo
- **Dataset DOI / Link:** <https://doi.org/10.5281/zenodo.21805403>

**Requirement 3: Copyright and Logo Issues**

**Comment:** “Questionnaire used for data collection.docx” contains branding/a logo. We ask that you please remove or replace it.

- **Response:** We apologize for this oversight.
- **Action taken:** We have completely removed the institutional branding and logo from the questionnaire file. The updated clean version of "Questionnaire used for data collection.docx” has been re-uploaded.

**Requirement 4: Ethics Statement Location**

**Comment:** Please ensure that your Ethics Statement is available in its entirety at the beginning of your Methods section, under a subheading 'Ethics Statement'*.*

- **Response:** Thank you for this structural guidance.
- **Action taken:** We have moved the complete Ethics Statement to the very beginning of the Methods section under the explicit subheading **‘Ethics Statement’**.

**Requirement 5: Supporting Information Legends**

**Comment:** Please add a full list of legends for your Supporting Information files after the references list.

- **Response:** Done.
- **Action taken:** A dedicated “Supporting Information” section with full explanatory legends for all uploaded supplementary files has been added immediately following the references list (Page [29], Lines [514-520]).

**Part 2: Additional Editor Comments**

**General & Title Changes**

**Comment 1:** The title should be focused on the area where the study took place to avoid providing an inflated title and misleading the reader.

- **Response:** We agree completely.
- **Action taken:** We have revised the title to explicitly state the localized setting of the study (Page [1], Lines [1-2]).

**Revised Title:** “School-based Prevalence and Factors Associated with Depressive symptoms among Secondary School Adolescents in Mahagadhimai Municipality, Nepal”

**Comment 2:** The CES-D-20 is a screening tool of the depressive symptoms and not depression. Please correct this throughout the entire document.

- **Response:** This is an excellent point. We acknowledge that the CES-D measures symptomatology rather than clinical diagnoses.
- **Action taken:** We have systematically updated the terminology throughout the entire text (including the title, abstract, results, and discussion) from “depression” to **“depressive symptoms”.**

**Introduction**

**Comment 3:** It would be better if you integrate a conceptual framework that guides the study.

- **Response:** Thank you for this suggestion, which provides strong theoretical grounding to our work.
- **Action taken:** We have integrated the conceptual framework into the Introduction section to guide the study’s exploration of individual and environmental psychosocial factors (Page [5-6], Lines [95-100]).

**Comment 4:** Explain why the prevalence of depression differs between countries. This would provide an analytical rather than descriptive aspect of the literature review.

- **Response:** We have transitioned this section to be more analytical.
- **Action taken:** We added text expanding on the structural, cultural and methodology differences (e.g., economic variations, varying thresholds of mental health stigma and distinct diagnostic cut-offs) that explain why the prevalence of depressive symptoms fluctuates between nations (Page [4], Lines [91-96]).

**Comment 5:** The rationale of the study is very briefly highlighted. You have to provide more evidence about the gaps in the literature and significance of the study within its context, Mahagadhimai Municipality, Nepal.

- **Response:** We have deepened the contextual rationale.
- **Action taken:** We have expanded the Introduction to explain that Mahagadhimai Municipality is a rural/semi-urban area undergoing rapid socio-economic shifts where adolescent mental health remains highly under-researched compared to metropolitan hubs like Kathmandu. We detailed specific regional challenges, such as limited mental health infrastructure and localized cultural pressures, to emphasize the gap our study fills (Page [4], Lines [84-91]).

**Methods**

**Comment 6:** Justify why the sample was limited only to adolescents in grades 11 and 12, attending four public schools. Why the private schools were excluded from the study?

- **Response:** We have provided clear academic, logistical, and contextual justifications for limiting our sample to Grades 11 and 12 public school students in the municipality.
- **Action taken:** We updated the Methods section to explain that Grades 11 and 12 represent a high-stress academic transition phase involving national board examinations and future career decisions. Furthermore, we clarified that private schools were excluded because private institutions in Mahagadhimai Municipality either had no active enrollments in Grades 11 and 12 at the time of data collection or had suspended their operational secondary programs due to insufficient student numbers. This institutional context has also been explicitly noted in the Limitations in the Discussion (Page [6], Lines [125-131]).

**Comment 7:** You said that all four public secondary schools in Mahagadhimai Municipality that provided Grades 11 and 12 were included through a census approach. What do you mean by census, as these are the only four schools?

- **Response:** We realize our initial description was ambiguous, and we have rewritten this section to clearly explain our sampling design.
- **Action taken:** We clarified in the Methods section that an **institutional census** was applied at the school level meaning all four functional public secondary schools offering Grades 11 and 12 in the municipality were included. We then applied a **multistage proportional stratified random sampling method** at the student level. Students were stratified by school, grade (11 vs. 12), and academic stream (Management vs. Education), with sample sizes allocated proportionally based on official enrollment registers, followed by simple random sampling within each stratum. (Page [7], Line [130-138]).

**Comment 8:** The sample is limited to a particular category of the population in a particular area, which limits the generalisation of findings. This limitation should be added in the discussion.

- **Response:** We completely agree with this assessment.
- **Action taken:** We added an explicit statement to the **Limitations** section of the Discussion, stating that because our sample was drawn exclusively from functional public secondary schools in Mahagadhimai Municipality, the findings cannot be generalized to out-of-school youth, students in private academies, or adolescents residing in urban metropolitan areas.

**Comment 9:** “academic faculty” is confusing for the non-Nepalese readers.

- **Response:** Thank you for pointing out this localized terminology.
- **Action taken:** We have changed “academic faculty” to **“academic stream”** (e.g., Science, Management, Humanities) to ensure clarity for an international audience.

**Comment 10:** The (CES-D-20) is the only described tool and all the others are neglected (BSMAS-6), (MSPSS-12) and (RES-10). No evidence of validity or reliability is provided of the academic stress measurement. Help-seeking variables are simplistic (single-item measures).

- **Response:** We appreciate this rigorous critique of our measurement descriptions.
- **Action taken:**
  - We expanded the Methods section to fully describe the **BSMAS-6** (Bergen Social Media Addiction Scale), **MSPSS-12** (Multidimensional Scale of Perceived Social Support), and **RES-10** (Rosenberg Self-Esteem Scale), providing their scoring ranges and interpretations.
  - We added previous validation data/Cronbach's alpha values for these tools within the Nepalese/South Asian context, as well as the calculated Cronbach's alpha from our own dataset.
  - We clarified that the academic stress and single-item help-seeking variables (sharing with parents/friends) were exploratory single-item indicators, explicitly stating this limitation in the text.

**Statistical Analysis & Results**

**Comment 11:** Include in multivariable analysis all variables with p < 0.25 in bivariate analysis to account with the biggest number of potential confounders... Family support was significant in bivariate analysis but not included in the multivariate analysis model.

- **Response:** We have re-run our statistical models based on this standard epidemiology practice.
- **Action taken:** We re-analyzed our data, entering all variables with P < 0.25 from the bivariate analysis (including family support) into the final multivariable logistic regression model. Tables [4] and the corresponding text in the Results and Discussion sections have been updated to reflect these revised, confounder-adjusted estimates.

**Comment 12:** Justify why self-esteem, social support and social media use were collapsed into categories, which limit their power. Why in the regression model, you collapsed the support into low + moderate categories?

- **Response:** We have provided our methodological justification in the text.
- **Action taken:** We explained in the Statistical Analysis section that these variables were collapsed to simplify interpretation for public health interventions (e.g., targeting a high-risk group vs. low/moderate risk) and to avoid structural sparsity in specific cross-tabulations. We justified combining low and moderate support because initial assessments showed their risk profiles for depressive symptoms were statistically similar compared to the high-support reference group.

**Comment 13:** Depression prevalence appears lower among those who keep problems to themselves than among those who do not. Yet the discussion interprets the opposite.

- **Response:** We thank the editor for highlighting this observation and have ensured our narrative matches our statistical tables.
- **Action taken:** We updated the Results and Discussion sections to precisely match our data: adolescents who **did not** try to solve problems on their own without sharing had four times higher odds of depressive symptoms (AOR = 4.09, 95% CI: 2.19-7.64). In the revised Discussion (Page [18], Lines [238–241]), we explained that self-reliant problem-solving in this context may reflect higher baseline emotional autonomy and resilience, whereas those experiencing severe distress may be more likely to seek external help and share problems.

**Comment 14:** 69.5% were ≥18 years old. For grades 11–12 this seems somewhat high and requires explanation. Table 1 states that the Median (min-max) age is 16–23. But this is not a median.

- **Response:** We agree that this age distribution requires contextualization and the reporting format was flawed.
- **Action taken:**
  - We corrected Table 1 to correctly label the metric as **“Median (IQR)”** or **“Range: 16–23”**.
- We added an explanatory paragraph in the Results section (Page [10], Lines [189–191]) contextualizing why 69.5% of students were aged $\geq18$ years. In rural public schools across Madhesh Province, older age distributions in Grades 11–12 are widely prevalent due to:
- **Board Exam Delays:** Frequent grade repetition following failures or re-examination attempts in the Secondary Education Examination (SEE / Grade 10 national board exams).
- **Academic Interruptions:** Temporary dropouts or pauses in schooling to assist families with seasonal farming, or domestic obligations before re-enrolling in higher secondary education.

**Discussion & Conclusion**

**Comment 15:** The discussion is descriptive rather than analytical with many redundant ideas. Avoid the overuse of predictors as this is a cross-sectional study. Family support lost significance in the adjusted model this deserves discussion.

- **Response:** We appreciate this constructive feedback. We have thoroughly restructured the Discussion section to shift from a descriptive summary to a deeper, more analytical interpretation of our findings.
- **Action taken:**
  - We streamlined the section to eliminate redundant comparisons and focus on an analytical interpretation of our findings within the socio-cultural fabric of Nepal.
  - We replaced causal jargon like "predictors" or "risk factors" with associative phrases like **“associated factors”**, **“correlates”**, or **“independent associations”**.
- We added an in-depth analytical paragraph explaining why family support lost statistical significance in the adjusted model. We discussed how this association may be attenuated when adjusting for robust concurrent variables like peer support or self-esteem, pointing to potential mediating pathways that warrant future longitudinal inquiry (Page [22], Lines [322–329]).

**Comment 16:** The conclusion implies intervention effectiveness which is not an accurate terminology for a cross-sectional study.

- **Response:** We appreciate this vital correction. We have revised our conclusion to align strictly with our study design.
- **Action taken:** The Conclusion section has been updated to remove any claims of direct intervention effectiveness. Instead, it frames academic stress, problematic social media use, social support, help-seeking behaviors, and self-esteem as potentially modifiable factors that warrant consideration in school-based mental health promotion. It explicitly acknowledges the study's cross-sectional design and notes that longitudinal and experimental research is required to evaluate whether programs targeting these factors can improve adolescent emotional well-being (Page [24], Lines [348–354]).

**Part 3: Reviewer #1 Comments (Dr. Akhilesh Srivastava)**

**Comment 1:** The cross-sectional design limits causal inference, yet the manuscript frequently uses causal terminology such as “predictors” and “risk factors.”

- **Response:** We acknowledge this limitation, which matches the Editor's comment.
- **Action taken:** As stated above, all instances of causal language (“predictors”, “causes”, “risk factors”) have been expunged and substituted with terms reflecting statistical correlation, such as “associated factors” or “correlates.”

**Comment 2:** Clarification is also needed regarding variable coding and interpretation, particularly for help-seeking behaviors. The rationale for the CES-D cutoff score should be better justified.

- **Response:** Thank you for bringing attention to these parameters.
- **Action taken:**
  - We clarified that help-seeking behaviors (e.g., sharing problems with parents/friends, self-reliant problem-solving) were assessed using **exploratory single-item indicators** with dichotomous coding (e.g., “Yes” vs. “No”).
- We expanded the rationale for our chosen CES-D cut-off score (>18) citing validated studies and literature that demonstrate its optimal sensitivity and specificity for identifying depressive symptomatology in Nepalese adolescent cohorts (Page [8], Lines [155–159]).

**Comment 3:** Important confounding variables were not assessed, limiting interpretability. Additionally, the manuscript requires considerable English language editing and reduction of repetitive discussion content.

- **Response:** We agree with these constructive points.
- **Action taken:**
  - We have explicitly listed unmeasured confounders (such as family history of mental illness, exact household socioeconomic status metrics, or recent negative life events) in our Limitations section.
  - The manuscript has undergone extensive English language proofreading to improve readability, flow and grammatical accuracy. Repetitive segments within the discussion have been synthesized or deleted.

We believe that these extensive improvements make our manuscript highly suitable for publication in PLOS Global Public Health. Thank you once again for your guidance.

Sincerely,

Harikishor Yadav
Corresponding Author
School of Health and Allied Sciences, Pokhara University

hariyadavph@gmail.com
