# Supplementary material for: School-based prevalence and factors associated with depressive symptoms among secondary school adolescents in Mahagadhimai Municipality, Nepal
Source: PLOS Glob Public Health. 2026 Sep 9;6(9):e0007225. doi: 10.1371/journal.pgph.0007225 (PMC13557374; doi:10.1371/journal.pgph.0007225)
Supplement: Peer review history

# Author comment: Author response to Decision Letter 0

**DOI:** 10.1371/journal.pgph.0007225.r001

20 Apr 2026

Attachment Submitted filename: Response letter.docx

# Peer review: Decision Letter 0

**DOI:** 10.1371/journal.pgph.0007225.r002

**Authors:** Mathilde Azar

23 Jun 2026

PGPH-D-26-01342

“School-based Prevalence and Factors Associated with Depression among Secondary School Adolescents in Nepal”

PLOS Global Public Health

Dear Dr. Yadav,

Thank you for submitting your manuscript to PLOS Global Public Health. After careful consideration, we feel that it has merit but does not fully meet PLOS Global Public Health’s publication criteria as it currently stands. Therefore, we invite you to submit a revised version of the manuscript that addresses the points raised during the review process.

Please submit your revised manuscript by Aug 22 2026 11:59PM. If you will need more time than this to complete your revisions, please reply to this message or contact the journal office at globalpubhealth@plos.org. When you're ready to submit your revision, log on to https://www.editorialmanager.com/pgph/ and select the 'Submissions Needing Revision' folder to locate your manuscript file.

Please include the following items when submitting your revised manuscript:

- A letter that responds to each point raised by the editor and reviewer(s). You should upload this letter as a separate file labeled 'Response to Reviewers'.
- A marked-up copy of your manuscript that highlights changes made to the original version. You should upload this as a separate file labeled 'Revised Manuscript with Track Changes'.
- An unmarked version of your revised paper without tracked changes. You should upload this as a separate file labeled 'Manuscript'.

Guidelines for resubmitting your figure files are available below the reviewer comments at the end of this letter.

As the corresponding author, your ORCID iD is verified in the submission system and will appear in the published article. PLOS supports the use of ORCID, and we encourage all coauthors to register for an ORCID iD and use it as well. Please encourage your coauthors to verify their ORCID iD within the submission system before final acceptance, as unverified ORCID iDs will not appear in the published article. *Only*  the individual author can complete the verification step; PLOS staff *cannot*  verify ORCID iDs on behalf of authors.

We look forward to receiving your revised manuscript.

Kind regards,

Mathilde Azar, PhD

Academic Editor

PLOS Global Public Health

**Journal Requirements:**

1.  Please provide a/amend your detailed Financial Disclosure statement. This is published with the article. It must therefore be completed in full sentences and contain the exact wording you wish to be published.

1. Please clarify all sources of funding (financial or material support) for your study. List the grants (with grant number) or organizations (with url) that supported your study, including funding received from your institution.

2. State the initials, alongside each funding source, of each author to receive each grant.

3. State what role the funders took in the study. If the funders had no role in your study, please state: “The funders had no role in study design, data collection and analysis, decision to publish, or preparation of the manuscript.”

4. If any authors received a salary from any of your funders, please state which authors and which funders.

If you did not receive any funding for this study, please simply state: “The authors received no specific funding for this work.”

2. We note that you have indicated that there are restrictions to data sharing for this study. For studies involving human research participant data or other sensitive data, we encourage authors to share de-identified or anonymized data. However, when data cannot be publicly shared for ethical reasons, we allow authors to make their data sets available upon request. For information on unacceptable data access restrictions, please see http://journals.plos.org/plosone/s/data-availability#loc-unacceptable-data-access-restrictions.

Before we proceed with your manuscript, please address the following prompts:

a) If there are ethical or legal restrictions on sharing a de-identified data set, please explain them in detail (e.g., data contain potentially identifying or sensitive patient information, data are owned by a third-party organization, etc.) and who has imposed them (e.g., a Research Ethics Committee or Institutional Review Board, etc.). Please also provide contact information for a data access committee, ethics committee, or other institutional body to which data requests may be sent.

b) If there are no restrictions, please upload the minimal anonymized data set necessary to replicate your study findings to a stable, public repository and provide us with the relevant URLs, DOIs, or accession numbers. Please see http://www.bmj.com/content/340/bmj.c181.long for guidelines on how to de-identify and prepare clinical data for publication. For a list of recommended repositories, please see https://journals.plos.org/plosone/s/recommended-repositories. You also have the option of uploading the data as Supporting Information files, but we would recommend depositing data directly to a data repository if possible.

Please update your Data Availability statement in the submission form accordingly.

3. Some material included in your submission may be copyrighted. According to PLOS’s copyright policy, authors who use figures or other material (e.g., graphics, clipart, maps) from another author or copyright holder must demonstrate or obtain permission to publish this material under the Creative Commons Attribution 4.0 International (CC BY 4.0) License used by PLOS journals. Please closely review the details of PLOS’s copyright requirements here: PLOS Licenses and Copyright. If you need to request permissions from a copyright holder, you may use PLOS's Copyright Content Permission form.

Please respond directly to this email or email the journal office and provide any known details concerning your material's license terms and permissions required for reuse, even if you have not yet obtained copyright permissions or are unsure of your material's copyright compatibility.

Potential Copyright Issues:

a. “Questionnaire used for data collection.docx” contains branding/a logo. We are not permitted to publish this under our CC-BY 4.0 license, even with permission. We ask that you please remove or replace it.

4. Please ensure that your Ethics Statement is available in its entirety at the beginning of your Methods section, under a subheading 'Ethics Statement'.

5. We have noticed that you have uploaded Supporting Information files, but you have not included a list of legends. Please add a full list of legends for your Supporting Information files after the references list.

If the reviewer comments include a recommendation to cite specific previously published works, please review and evaluate these publications to determine whether they are relevant and should be cited. There is no requirement to cite these works unless the editor has indicated otherwise.

**Additional Editor Comments (if provided):**

PGPH-D-26-01342 comments

The topic is interesting and relevant and findings have potential implications on the mental wellbeing of adolescents. The below comments should be taken into consideration to improve the quality of the manuscript to become ready for publication.

The title should be focused on the area where the study took place to avoid providing an inflated title and misleading the reader.

The CES-D is a screening tool of the depressive symptoms and not depression. Please correct this throughout the entire document.

Introduction

It would be better if you integrate a conceptual framework that guides the study.

Explain why the prevalence of depression differs between countries. This would provide an analytical rather than descriptive aspect of the literature review.

The rationale of the study is very briefly highlighted. You have to provide more evidence about the gaps in the literature and significance of the study within its context, Mahagadhimai Municipality, Nepal.

Methods

Justify why the sample was limited only to adolescents in grades 11 and 12, attending four public schools.

You said that all four public secondary schools in Mahagadhimai Municipality that provided Grades 11 and 12 were included through a census approach. What do you mean by census, as these are the only four schools?

The sample is limited to a particular category of the population in a particular area, which limits the generalisation of findings. This limitation should be added in the discussion.

Why the private schools were excluded from the study?

“academic faculty” is confusing for the non-Nepalese readers.

The (CES-D-20) is the only described tool and all the others are neglected (BSMAS-6), (MSPSS-12) and (RES-10).

No evidence of validity or reliability is provided of the academic stress measurement.

Help-seeking variables are simplistic. Variables such as share problems with parents and share problems with friends appear to be single-item measures. There is no evidence of validity or reliability.

Statistical analysis

Include in multivariable analysis all variables with p < 0.25 in bivariate analysis to account with the biggest number of potential confounders.

Family support was significant in bivariate analysis but not included in the multivariate analysis model.

Justify why self-esteem, social support, and social media use were collapsed into categories, which limit their power.

Results

Depression prevalence appears lower among those who keep problems to themselves than among those who do not. Yet the discussion interprets the opposite.

69.5% were ≥18 years old.

For grades 11–12 this seems somewhat high and requires explanation

Table 1 states that the Median (min-max) age is 16–23. But this is not a median.

Justify why in the regression model, you collapsed the support into low + moderate categories

Discussion

The discussion is descriptive rather than analytical with many redundant ideas.

Avoid the overuse of predictors a this is a cross-sectional study

It is very common to

Family support lost significance in the adjusted model. This is theoretically important and deserves discussion.

The conclusion implies intervention effectiveness which is not an accurate terminology for a cross-sectional study.

[Note: HTML markup is below. Please do not edit.]

Reviewers' comments:

Reviewer's Responses to Questions

**Comments to the Author**

1. Does this manuscript meet PLOS Global Public Health’s publication criteria?

Reviewer #1: Yes

**********

2. Has the statistical analysis been performed appropriately and rigorously?

Reviewer #1: Yes

**********

3. Have the authors made all data underlying the findings in their manuscript fully available (please refer to the Data Availability Statement at the start of the manuscript PDF file)?

The PLOS Data policy

Reviewer #1: Yes

**********

4. Is the manuscript presented in an intelligible fashion and written in standard English?

Reviewer #1: Yes

**********

**Reviewer #1:**  This manuscript investigates depressive symptoms and associated psychosocial factors among secondary school adolescents in Nepal using validated instruments and appropriate statistical methods. The study addresses a significant public health issue and contributes valuable regional evidence. Major strengths include the use of standardized scales, stratified sampling, and practical implications for school mental health interventions.

However, substantial revisions are needed. The cross-sectional design limits causal inference, yet the manuscript frequently uses causal terminology such as “predictors” and “risk factors.” Clarification is also needed regarding variable coding and interpretation, particularly for help-seeking behaviors. The rationale for the CES-D cutoff score should be better justified. Important confounding variables were not assessed, limiting interpretability. Additionally, the manuscript requires considerable English language editing and reduction of repetitive discussion content.

Overall, the manuscript has merit but requires revision before it is suitable for publication.

**********

what does this mean?). If published, this will include your full peer review and any attached files.

**Do you want your identity to be public for this peer review?**  If you choose “no”, your identity will remain anonymous but your review may still be made public.

For information about this choice, including consent withdrawal, please see our Privacy Policy

Reviewer #1: **Yes:**  Dr Akhilesh Srivastava

**********

[NOTE: If reviewer comments were submitted as an attachment file, they will be attached to this email and accessible via the submission site. Please log into your account, locate the manuscript record, and check for the action link "View Attachments". If this link does not appear, there are no attachment files.]

**Figure Resubmissions:**

After uploading your figures to PLOS’s NAAS tool - https://ngplosjournals.pagemajik.ai/artanalysis, NAAS will process the files provided and display the results in the "Uploaded Files" section of the page as the processing is complete. If the uploaded figures meet our requirements (or NAAS is able to fix the files to meet our requirements), the figure will be marked as "fixed" above. If NAAS is unable to fix the files, a red "failed" label will appear above. When NAAS has confirmed that the figure files meet our requirements, please download the file via the download option, and include these NAAS processed figure files when submitting your revised manuscript.

Attachment Submitted filename: comments_PGPH-D-26-01342.docx

# Author comment: Author response to Decision Letter 1

**DOI:** 10.1371/journal.pgph.0007225.r003

12 Aug 2026

Attachment Submitted filename: Response to Reviewers.docx

# Editor report: Decision Letter 1

**DOI:** 10.1371/journal.pgph.0007225.r004

**Authors:** Mathilde Azar

17 Aug 2026

“School-based Prevalence and Factors Associated with Depressive symptoms among Secondary School Adolescents in Mahagadhimai Municipality, Nepal”

PGPH-D-26-01342R1

Dear Mr. Yadav,

We are pleased to inform you that your manuscript '“School-based Prevalence and Factors Associated with Depressive symptoms among Secondary School Adolescents in Mahagadhimai Municipality, Nepal”' has been provisionally accepted for publication in PLOS Global Public Health.

Before your manuscript can be formally accepted you will need to complete some formatting changes, which you will receive in a follow up email. A member of our team will be in touch with a set of requests.

Please note that your manuscript will not be scheduled for publication until you have made the required changes, so a swift response is appreciated.

IMPORTANT: The editorial review process is now complete. PLOS will only permit corrections to spelling, formatting or significant scientific errors from this point onwards. Requests for major changes, or any which affect the scientific understanding of your work, will cause delays to the publication date of your manuscript.

If your institution or institutions have a press office, please notify them about your upcoming paper to help maximize its impact. If they'll be preparing press materials, please inform our press team as soon as possible -- no later than 48 hours after receiving the formal acceptance. Your manuscript will remain under strict press embargo until 2 pm Eastern Time on the date of publication. For more information, please contact globalpubhealth@plos.org.

Thank you again for supporting Open Access publishing; we are looking forward to publishing your work in PLOS Global Public Health.

Best regards,

Mathilde Azar, PhD

Academic Editor

PLOS Global Public Health

***********************************************************

No additional comments.

Reviewer Comments (if any, and for reference):
